# Early evaluation of radiation-induced parotid damage in patients with nasopharyngeal carcinoma by T2 mapping and mDIXON Quant imaging: initial findings

**DOI:** 10.1186/s13014-018-0970-9

**Published:** 2018-02-08

**Authors:** Nan Zhou, Chen Chu, Xin Dou, Weibo Chen, Jian He, Jing Yan, Zhengyang Zhou, Xiaofeng Yang

**Affiliations:** 1 Department of Radiology, Nanjing Drum Tower Hospital, The Affiliated Hospital of Nanjing University Medical School, Nanjing, 210008 China; 2 Philips Healthcare, Shanghai, 200233 China; 3 The Comprehensive Cancer Centre of Nanjing Drum Tower Hospital, Clinical College of Nanjing Medical University, Nanjing, 210008 China; 4 Department of Radiation Oncology and Winship Cancer Institute, Emory University, Atlanta, GA 30322 USA

**Keywords:** Radiation-induced parotid damage, Magnetic resonance imaging, T2 mapping, mDIXON Quant imaging, Quantitative evaluation

## Abstract

**Background:**

Radiation-induced parotid damage is a common complication in patients with nasopharyngeal carcinoma (NPC) treated with radiotherapy to head and neck region, which severely reduce the life quality of those patients. The aim of this study was to early evaluate the changes of irradiated parotid glands with T2 mapping and mDIXON Quant imaging.

**Methods:**

Forty-one patients with NPC underwent conventional magnetic resonance imaging for nasopharynx and neck, and T2 mapping and mDIXON Quant imaging for bilateral parotid glands within 2 weeks before radiotherapy (pre-RT), 5 weeks after the beginning of radiotherapy (mid-RT), and 4 weeks after radiotherapy (post-RT). Parotid volume, T2 values, fat fraction (FF) values, and mean radiation dose were recorded and analyzed.

**Results:**

From pre-RT to mid-RT, parotid volume decreased (atrophy rate, 27.0 ± 11.5%), while parotid T2 and FF values increased (change rate, 6.0 ± 6.2% for T2 value and 9.1 ± 9.9% for FF value) significantly. From mid-RT to post-RT, parotid T2 value continuously increased (change rate, 4.6 ± 7.7%), but parotid FF value decreased (change rate, − 9.9 ± 18.2%) significantly. Change rate of parotid T2 value significantly correlated with parotid atrophy rate from pre-RT to post-RT (r = 0.313, *P* = 0.027). Multiple linear regression analysis showed that parotid T2 value (standardized coefficient [SC] = − 0.259, *P* = 0.001) and FF value (SC = − 0.320, *P* = 0.014) negatively correlated with parotid volume, while parotid T2 value positively correlated with MR scan time point (SC = 0.476, *P* = 0.001) significantly. Parotid T2 and FF values showed excellent reproducibility (intraclass correlation coefficient, 0.935–0.992).

**Conclusions:**

T2 mapping and mDIXON Quant imaging is useful for noninvasive evaluation of radiation-induced parotid damage.

## Background

Patients with nasopharyngeal carcinoma (NPC) always suffer from radiation-induced xerostomia, dysphagia, and even dental caries, which severely reduce their life quality [1]. In recent years, intensity-modulated radiotherapy (IMRT) has been applied to treat NPC, which helps to alleviate radiation-induced parotid damage [2]. Due to their sensitivity to radiation [3], parotid glands cannot entirely escape radiation-induced damage even with IMRT [4]. Since parotid glands are the largest salivary glands producing 60%–65% of the whole saliva [5], early evaluation of radiation-induced parotid damage would facilitate a timely adjustment of treatment scheme to alleviate the damage of parotid glands.

Degree of xerostomia can be clinically evaluated with Radiation Therapy Oncology Group (RTOG) criteria [6], which mainly relies on subjective symptoms. Biopsy can bring microstructural information of irradiated parotid glands [7], but it is invasive and unsuitable for clinical practice. Scintigraphy can quantitatively assess the changes of parotid function [8], which has a disadvantage of extra radiation exposure. Magnetic resonance (MR) imaging is a noninvasive modality to evaluate radiation-induced parotid damage with a high soft tissue contrast [9].

Previous studies confirmed the glandular edema and fatty replacement in irradiated parotid glands [10, 11]. Tissue edema can be quantitatively evaluated by T2 mapping, since T2 relaxation time extends when free water increases [12]. Fat content in tissues can be quantified with fat fraction (FF) generated by mDIXON Quant imaging [13]. The feasibility of T2 mapping and mDIXON Quant imaging in parotid glands has been confirmed in healthy volunteers [14, 15]. Nevertheless, application of those MR imaging has never been reported in evaluating radiation-induce parotid damage during radiotherapy.

Therefore, the primary purpose of this study was to early evaluate the dynamic changes of radiation-induced parotid damage with T2 mapping and mDIXON Quant imaging. Correlations between parotid T2 or FF values and parotid atrophy rate or mean radiation dose were also investigated.

## Methods

### Patients

This study was approved by the institutional review board. After providing written informed consents, 51 patients was enrolled prospectively. The inclusion criteria were: (1) with a biopsy confirmed diagnosis of NPC; (2) scheduled to undergo IMRT; (3) willing to receive MR evaluation and follow-ups in our hospital. The exclusion criteria included: (1) with a history of radiotherapy to the head and neck region (*n* = 0); (2) with parotid gland diseases, such as Sjögren’s syndrome (*n* = 1); (3) with MR contraindications, such as cardiac pacemaker or cochlear implants (*n* = 0); (4) unable to accomplish the whole course of MR follow-ups (*n* = 7); (5) with geometric distortion or physiologic motion on MR images (*n* = 2). Hence, 41 patients with NPC served as our study cohort (male, 29; female, 12; age range, 21–70 years; mean age, 49.1 ± 11.5 years).

### Chemoradiotherapy and xerostomia degree assessment

The chemoradiotherapy scheme for NPC patients has been described in a previous article [16]. All patients received MR scans at three time points: pre-RT, within 2 weeks before radiotherapy; mid-RT, 5 weeks after the beginning of radiotherapy; and post-RT, 4 weeks after radiotherapy. Bilateral parotid glands were analyzed separately due to different radiation doses received by them. Parotid mean radiation dose was obtained from the treatment planning systems of TomoTherapy HiArt (TomoTherapy, Madison, WI, USA) and Pinnacle^3^ (Philips Medical Systems, Fitchburg, WI, USA). The accumulated mean radiation dose of parotid gland at mid-RT (20.4 ± 2.7 Gy) or post-RT (29.2 ± 3.3 Gy) was also calculated. The mean radiation dose of parotid gland in each patient was under the constraint in our hospital (30–50 Gy for 50% of parotid volume).

One hour before MR scanning, the xerostomia degree of patients was assessed by a radiation oncologist (X.X., with 10 years’ experience in radiotherapy to the head and neck region) according to RTOG criteria [6]. The xerostomia degree was grade 0 in all patients at pre-RT, and increased to grade 1 (20 patients) or 2 (21 patients) at mid-RT. From mid-RT to post-RT, the xerostomia degree remained unchanged in 36 patients, and decreased from grade 2 to 1 in 4 patients, and increased from grade 1 to 2 in 1 patients.

### MR examination

All patients was required to fast for at least 2 h before MR scanning. A 3.0-T MR scanner (Ingenia, Philips Medical Systems, Best, the Netherlands) with a 16-channel head&neck phased-array coil was used. 32-channel Torso coil was added for mDIXON Quant imaging. The patient’s position was head first and supine. Among 41 patients, 22 patients underwent conventional MR and T2 mapping imaging (from May 2016 to January 2017), 6 patients underwent conventional MR, T2 mapping, and mDIXON Quant imaging (from January 2017 to March 2017), and the remaining 13 patients underwent conventional MR and mDIXON Quant imaging (from March 2017 to July 2017), respectively. The conventional MR imaging included fat-saturated T2-weighted imaging in coronal and transverse planes, T1-weighted imaging in coronal, sagittal, and transverse planes, and contrast enhanced fat-saturated T1-weighted imaging in coronal, sagittal, and transverse planes.

T1-weighted imaging was obtained by using turbo spin-echo (TSE) sequence, whose parameters were as follows: repetition time (TR) / echo time (TE) = 400–675 msec / 18 msec, field of view (FOV) = 22 cm, voxel size = 0.8 mm × 0.92 mm, matrix = 276 × 215, slice thickness = 5 mm, slices = 38, number of signals averaged (NSA) = 2. The scan duration was 2 min 27 s.

T2 mapping was obtained by using multi-slice multi-echo TSE sequence, whose parameters were as follows: TR / TE1 = 2109 msec / 17 msec, delta TE = 17 msec, echoes = 5, FOV = 22 cm, voxel size = 1.8 mm × 2.4 mm, matrix = 124 × 90, slice thickness = 4 mm, slices = 20, NSA = 1. The scan duration was 33 s.

mDIXON Quant imaging was obtained by using 3D fast field echo (FFE) sequence, whose parameters were as follows: TR / TE1 = 10 msec / 1.47 msec, delta TE = 1.2 msec, echoes = 6, FOV = 25 cm × 24 cm, voxel size = 1.2 mm × 1.2 mm, matrix = 208 × 201, slice thickness = 2.5 mm, slices = 64, NSA = 2. The scan duration was 2 min 7 s. A low flip angle of only 3° was applied to limit the T1 bias, and 6 echoes was used to correct for T2* effects.

### Image analysis

Measurements of MR parameters were performed by two radiologists (X.X. and X.X., with 7 and 15 years’ experience in MR imaging of head and neck region, respectively) independently. Both of them were blinded to the clinical data of patients. Parotid volume, T2, and FF values were measured on a workstation (Extended MR WorkSpace 2.6.3.5, Philips Medical Systems, Best, the Netherlands).

Each slice area of parotid gland was measured on T1-weighted image. Parotid volume was calculated by using the following equation: V = Σ S푖 × (ST + SG), where V is parotid volume, S푖 is each slice area, ST is slice thickness, and SG is slice gap. The atrophy rate of parotid gland was calculated by using the following equation: R_V_ = (V_pre_ − V_mid/post_) / V_pre_ × 100%, where R_V_ is the atrophy rate of parotid gland from pre-RT to mid-RT or post-RT, and V_pre_ and V_mid/post_ are parotid volume at pre-RT, mid-RT, and post-RT, respectively.

After acquisition of T2 mapping and mDIXON Quant sequence, T2 and FF maps were automatically calculated. The regions of interest (ROIs) were drawn within the largest three slices of parotid gland on T2-wighted image (T2 mapping sequence with TE = 51 msec) and FF map to include as much parotid parenchyma as possible excluding visible parotid ducts and retromandibular veins. The ROIs on T2-wighted image were manually copied to the corresponding T2 map to calculate T2 value. The T2 and FF values were defined as the averaged value of the largest three slices. The change rates of parotid T2 and FF values were calculated by using the following equation: R_Para_ = (Para_mid/post_ – Para_pre_)/Para_pre_ × 100%, where R_Para_ is the change rates of parotid T2 or FF values from pre-RT to mid-RT or post-RT, and Para_pre_ and Para_mid/post_ are the T2 or FF value of parotid gland at pre-RT, mid-RT, and post-RT, respectively.

The final T2 and FF values of parotid gland were recorded as the averaged value of the two radiologists’ measurements. With an interval of 12 weeks, parotid T2 and FF values were re-measured by the second radiologist to assess the intra-oberver reproducibility.

### Statistical analysis

Continuous data were shown as mean ± standard deviation. The dynamic changes of parotid volume, T2 and FF values were analyzed by using paired sample *t* test with Bonferroni correction. Two independent-samples *t* test was used to compare the change rates of parotid T2 or FF value between men and women. Pearson’s correlation test was used to detect correlations between the change rates of T2 or FF value and parotid atrophy rate, mean radiation dose, or age. Correlations among parotid T2 or FF value, parotid volume, and MR scan time point was detected by multiple linear regression analysis. Intraclass correlation coefficient (ICC) was used to evaluate the reproducibility of parotid T2 and FF values measurements. All statistical analyses were performed using SPSS 16.0 software (SPSS Inc., Chicago, IL, USA). A *P* value < 0.017 for paired sample *t* test with Bonferroni correction and a *P* value < 0.05 for other statistical analyses indicated a statistical significance.

## Results

### Dynamic changes of parotid volume, T2 and FF values during radiotherapy

**Fig. 1:**
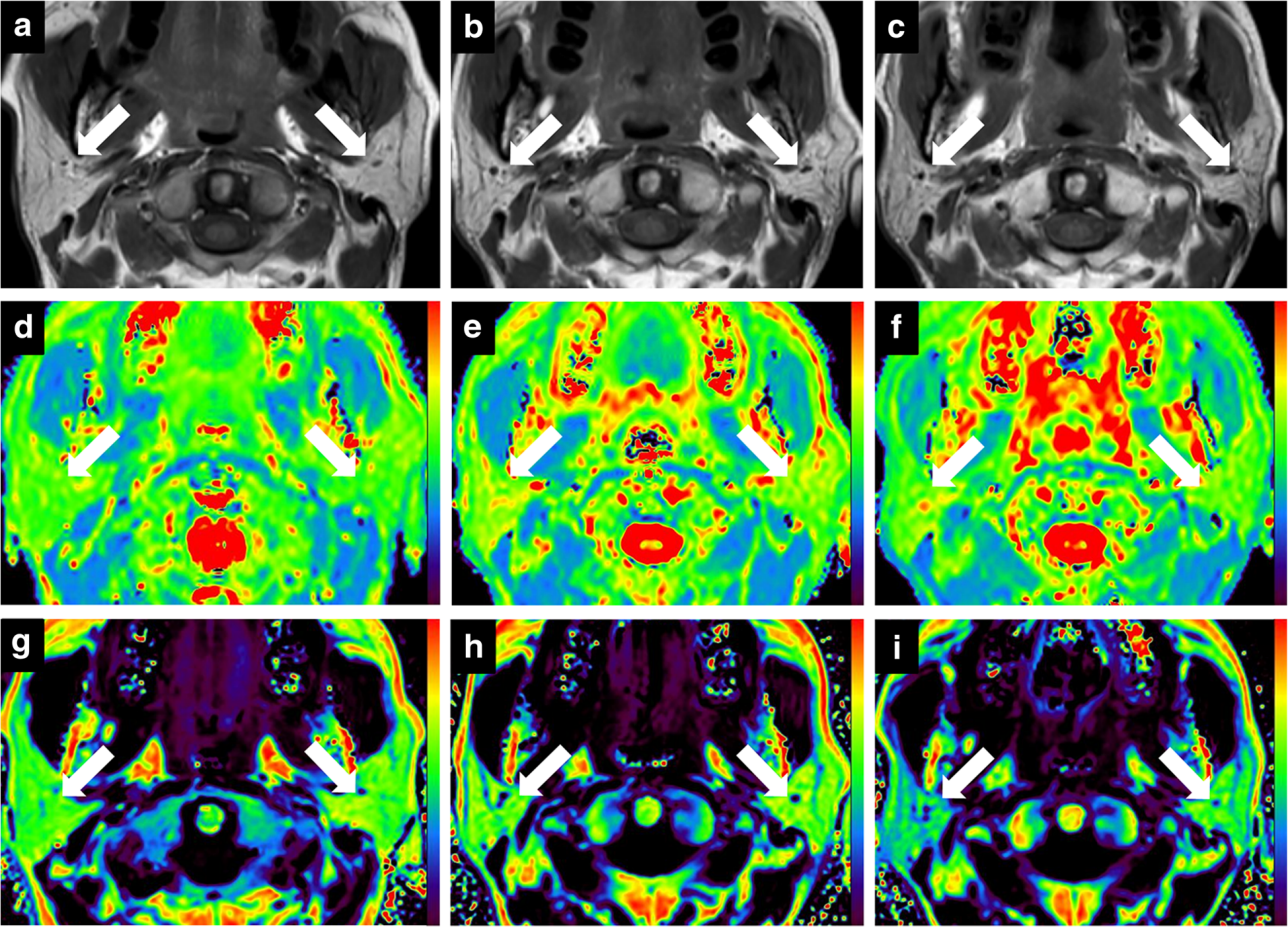
Dynamic changes of T1-weighted images (**a-c**), T2 maps (**d-f**), and fat fraction (FF) (**g-i**) maps of bilateral parotid glands (white arrows) in one patient with nasopharyngeal carcinoma (NPC) within 2 weeks before radiotherapy (pre-RT), 5 weeks after the beginning of radiotherapy (mid-RT), and 4 weeks after radiotherapy (post-RT), respectively. T1-weighted images show that right and left parotid volume are 30.1 cm^3^ and 30.2 cm^3^ (pre-RT, **a**), 21.9 cm^3^ and 20.1 cm^3^ (mid-RT, **b**), and 23.2 cm^3^ and 20.2 cm^3^ (post-RT, **c**), respectively. T2 maps show that right and left parotid T2 values are 70.9 msec and 65.8 msec (pre-RT, **d**), 75.4 msec and 75.1 msec (mid-RT, **e**), and 75.8 msec and 72.2 msec (post-RT, **f**), respectively. FF maps show that right and left parotid FF values are 54.9% and 57.4% (pre-RT, **g**), 59.0% and 57.3% (mid-RT, **h**), and 45.9% and 50.4% (post-RT, **i**), respectively

The dynamic changes of parotid volume, T2 and FF values are shown in Table 1. The T1-weighted images, T2 and FF maps of bilateral parotid glands in one patient with NPC are shown in Fig. 1.

**Table 1:** Dynamic changes of parotid MR parameters during radiotherapy

	pre-RT	mid-RT	post-RT
V (cm^3^)	27.2 ± 8.2	19.4 ± 5.5^a^	19.5 ± 5.7^a^
T2 (msec)	71.7 ± 5.2	76.0 ± 7.3^a^	79.4 ± 9.0^a,b^
FF (%)	38.2 ± 9.7	42.1 ± 10.3^a^	38.8 ± 13.5^b^

Note: *V* volume, *FF* fat fraction, *pre-RT* within 2 weeks before radiotherapy, *mid-RT* 5 weeks after the beginning of radiotherapy, *post-RT* 4 weeks after radiotherapy. ^a^ *P* < 0.017 compared with pre-RT and ^b^ *P* < 0.017 compared with mid-RT using paired sample *t* test with Bonferroni correlation

From pre-RT to mid-RT, parotid volume decreased significantly (atrophy rate, 27.0 ± 11.5%). However, no significant difference was found in parotid volume from mid-RT to post-RT. Hence, the overall atrophy rate of parotid volume from pre-RT to post-RT was 27.0 ± 10.8%.

From pre-RT to mid-RT, parotid T2 value increased significantly (change rate, 6.0 ± 6.2%), and continued to increase significantly from mid-RT to post-RT (change rate, 4.6 ± 7.7%). Hence, the overall change rate of parotid T2 value from pre-RT to post-RT was 10.8 ± 10.0%.

From pre-RT to mid-RT, parotid FF value increased significantly (change rate, 9.1 ± 9.9%), and then decreased significantly from mid-RT to post-RT (change rate, − 9.9 ± 18.2%). Hence, parotid FF value did not change significantly from pre-RT to post-RT.

From pre-RT to mid-RT or post-RT, no significant differences of the change rates of parotid T2 or FF value were found between men (6.1 ± 6.1% and 10.1 ± 11.0% for T2 and FF values at mid-RT, 11.5 ± 10.2% and - 7.4 ± 22.7% for T2 and FF values at post-RT, respectively) and women (5.4 ± 1.9% and 11.5 ± 4.5% for T2 and FF values at mid-RT, 8.0 ± 5.1% and 10.0 ± 5.8% for T2 and FF values at post-RT, respectively) (all *P* > 0.05).

### Correlations between parotid T2 or FF values and parotid atrophy rate or mean radiation dose

As shown in Table 2, the only significant correlation existed between the change rate of parotid T2 value and parotid atrophy rate from pre-RT to post-RT (r = 0.313, *P* = 0.027).

**Table 2:** Correlations between the change rates of parotid T2 or FF values and parotid atrophy rates or mean radiation dose during radiotherapy

	R_V_		Mean radiation dose	
	r	*P* value	r	*P* value
Mid-RT				
R_T2_	0.092	0.525	0.087	0.530
R_FF_	0.038	0.820	0.121	0.591
Post-RT				
R_T2_	0.313	0.027^a^	−0.106	0.444
R_FF_	−0.029	0.863	−0.419	0.052

Note: *Pre-RT* within 2 weeks before radiotherapy, *mid-RT* 5 weeks after the beginning of radiotherapy, *post-RT* 4 weeks after radiotherapy. R_V_, R_T2_ and R_FF_ are the change rates of parotid volume, T2 value and fat fraction (FF) compared with pre-RT, respectively. ^a^ *P* < 0.05 with Pearson’s correlation test

### Correlations among parotid T2 or FF value, parotid volume, and MR scan time point

Multiple linear regression analysis showed that parotid T2 value negatively correlated with parotid volume (standardized coefficient [SC] = − 0.259, *P* = 0.001), but positively correlated with MR scan time point (SC = 0.476, *P* < 0.001) significantly. Parotid FF value negatively correlated with parotid volume significantly (SC = − 0.320, *P* = 0.014), yet did not correlate with MR scan time point (SC = − 0.104, *P* = 0.417).

### Reproducibility of parotid T2 and FF value measurements

The intra- and inter-oberver ICCs of parotid T2 and FF values were 0.939 (95% confidence interval [CI], 0.916–0.955) and 0.935 (95% CI, 0.910–0.953) for T2 value, and 0.992 (95% CI, 0.985–0.996) and 0.984 (95% CI, 0.972–0.990) for FF value, respectively.

## Discussion

Parotid volume decreased significantly after radiotherapy with an atrophy rate of 27%, which might be due to the loss of acinar cells [10]. Houweling et al. reported a mean parotid volume shrinkage of 27% in patients with oropharyngeal cancer 6 weeks after radiotherapy [17], which was consistent with our result.

Parotid T2 value continuously increased during radiotherapy, which can be explained by the continuous aggravation of tissue edema [10]. Houweling et al. also reported a significant increase of parotid T2 signal intensity 6 weeks after radiotherapy [17], which was in line with our results. We also confirmed a positive correlation between the change rate of parotid T2 value and parotid atrophy rate from pre-RT to post-RT. A possible explanation is that the enlarged extracellular space due to the loss of acinar cells caused an accumulation of free water in parotid parenchyma [10]. It was reported that parotid shrinkage was significantly correlated with the decrease of saliva production in patients with head-and-neck cancer undergoing radiotherapy [18]. However, the correlation between the change rate of parotid T2 value and parotid atrophy rate was weak. A similar finding was obtained by a previous study, which also showed low correlations between changes in parotid IVIM MR parameters and parotid shrinkage (r = 0.336 and *P* = 0.023 for *ADC*; r = 0.357 and *P* = 0.018 for *ADClow*; r = 0.378 and *P* = 0.012 for *f*) [19]. Hence, such a low correlation indicated that the change of MR parameters in irradiated parotid glands might be involved with other factors such as heterogeneous distribution of parotid radiation dose and different radiation sensitivity of individual parotid gland.

The pre-RT parotid FF value of NPC patients (mean age, 47.8 years) was approximately 38.2% in this study. Kise et al. reported a mean parotid FF value of 29.4% in healthy volunteers (mean age, 37.6 years) at 3.0-T MR scanner, which was slightly lower than ours [13]. Chang et al. reported a positive correlation between parotid FF value and age in healthy adults [20], which might explain the difference between Kise et al.’s and our results.

Parotid FF value first increased and then decreased during radiotherapy. Multiple linear regression analysis revealed that early increase of FF value was mainly due to parotid atrophy from pre-RT to mid-RT. From mid-RT to post-RT, parotid volume remained unchanged, while glandular edema continuously aggravated. The FF value reflects the relative proton density ratio between fat signal and the sum of fat and water signals [15, 21], which might suggest that the decrease of parotid FF value was due to glandular edema from mid-RT to post-RT.

Parotid T2 and FF values showed excellent intra- and inter-observer reproducibility. Serai et al. reported an ICC of 0.992 for parotid FF value in healthy volunteers obtained from mDIXON Quant imaging at 3.0-T MR scanner [22], which was consistent with our findings.

Our study had several limitations. First, the sample size was relatively small, yet still larger than other investigations on radiation-induced parotid damage with MR imaging [17, 19]. Second, parotid gland biopsy was not performed due to its invasiveness. Third, long-term follow-ups was not performed. Evaluation of late fatty replacement in irradiated parotid glands should be further investigated.

## Conclusions

T2 mapping and mDIXON Quant imaging provided pathophysiological information of parotid gland during radiotherapy, which could serve as an objective and quantitative modality in evaluating early radiation-induced parotid damage and facilitate a timely adjustment of treatment scheme to alleviate the damage of parotid glands.

## Abbreviations

CI: Confidence interval
FF: Fat fraction
ICC: Intraclass correlation coefficient
IMRT: Intensity-modulated radiotherapy
mid-RT: 5 weeks after the beginning of radiotherapy
MR: Magnetic resonance
NPC: Nasopharyngeal carcinoma
post-RT: 4 weeks after radiotherapy
pre-RT: Within 2 weeks before radiotherapy
